# Intramyocardial dissecting haematoma with false pseudoaneurysm

**DOI:** 10.1093/ehjcr/ytag124

**Published:** 2026-02-18

**Authors:** Diana Isabel Katekaru-Tokeshi, Moisés Jiménez-Santos, Eric Kimura-Hayama

**Affiliations:** Service of Cardiology, Hospital Nacional Dos de Mayo, Miguel Grau avenue 13, 15003 Lima, Peru; Departament of Radiology, Service of Computed Tomography, Instituto Nacional de Cardiologia ‘Ignacio Chavez’, Juan Badiano 1, Col. Belisario Dominguez, Seccion XVI, Tlalpan, 14080 Mexico City, Mexico; CT Scanner Lomas Altas, Paseo de la reforma avenue 2608, Colonia Real de Lomas, Miguel Hidalgo, 11950 Mexico City, Mexico; Departament of Radiology, Service of Computed Tomography, Instituto Nacional de Cardiologia ‘Ignacio Chavez’, Juan Badiano 1, Col. Belisario Dominguez, Seccion XVI, Tlalpan, 14080 Mexico City, Mexico; CT Scanner Lomas Altas, Paseo de la reforma avenue 2608, Colonia Real de Lomas, Miguel Hidalgo, 11950 Mexico City, Mexico

## Case description

A 76-year-old man with dyspnoea of moderate efforts. Three months ago he had a myocardial infarction that was thrombolyzed 6 h after the onset of precordial pain at another institution. ECG showed Q waves in II, AVF, and from V_2_ to V_6_ leads.

**Figure 1:**
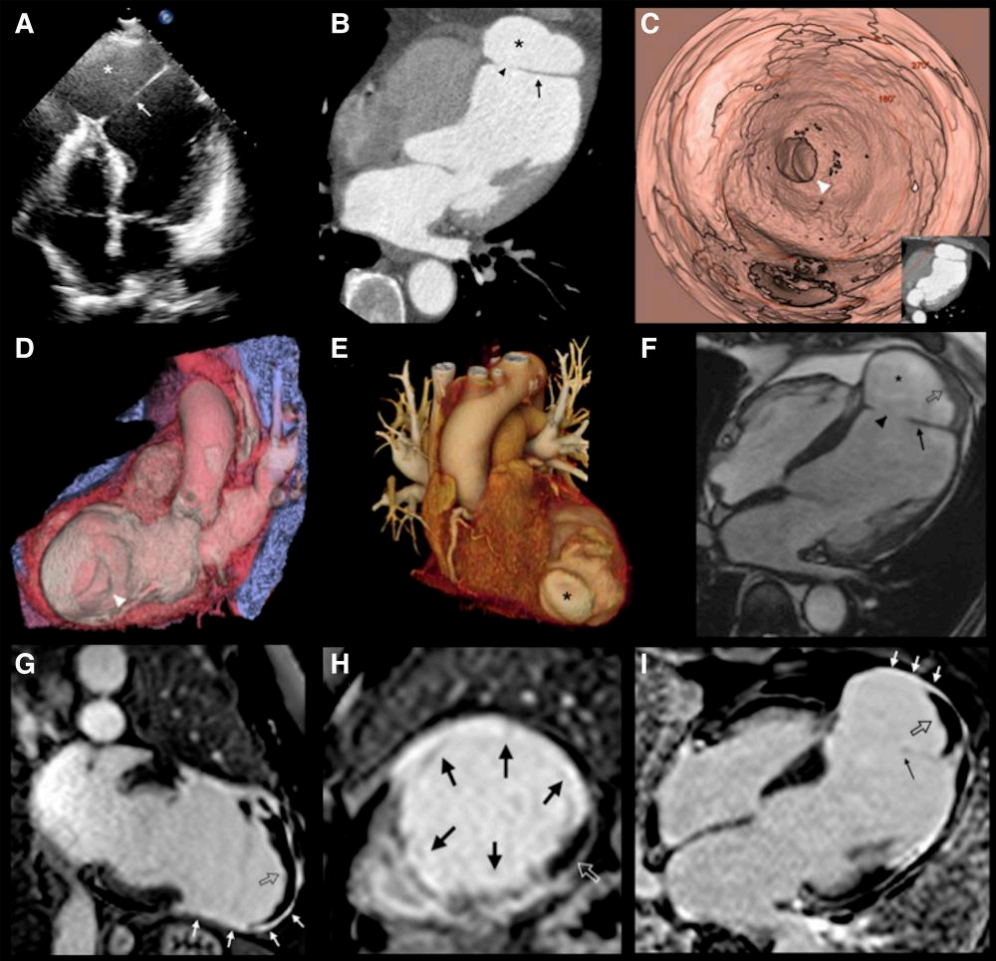
(*A*) Transthoracic echocardiography apical four-chamber (4C) view showing a dissection flap at the apical level (arrow) connecting the left ventricle with a large cavity (*). (*B*) An axial view of cardiac CT confirmed the defect of the myocardial wall (arrowhead) connecting the left ventricle with the cavity (*) previously described by TTE and apparently contained by the pericardium. A false pseudoaneurysm was considered. (*C-D*). Virtual CT ventriculoscopy and long axial 3D VR image with lateral cut showing the defect (arrowhead) in the myocardial wall communicating with the abnormal cavity of the false-pseudoaneurysm. (*E*) Cardiac CT VR reconstruction allowed the visualization of false-pseudoaneurysm from its surface (*). (*F*) CMR apical 4C view with cine showed the presence of thrombus (open arrow) within the false pseudoaneurysm (*), and the communication (arrowhead) through the thinned myocardial wall (arrow). (*G–I*). CMR Phase-Sensitive Inversion Recovery (PSIR) late gadolinium enhancement reconstructions involving the mid-segment of the anterior wall and apical segments (black arrows) and thrombus (open arrow) surrounded by enhancing overlying pericardium (white arrows).

Transthoracic echocardiography evidenced severe left ventricular (LV) systolic dysfunction (ejection fraction of 25%) with anterior severe hypokinesis and apical akinesia. Also it noted the presence of a thin and mobile rounded cavity at the apical level connecting the LV with a large cavity apparently contained by the pericardium, suggestive of a contained rupture (see *Figure 1A*, Supplementary material, *S1*). Coronary angiography reported proximal occlusion of the left anterior descending artery.

The cardiac CT demonstrated more clearly the defect in the myocardial wall communicating with the abnormal cavity compatible with a false pseudoaneurysm (Figure 1*B–E*). Cardiovascular MR revealed the presence of a laminar thrombus that was surrounded by enhancing overlying pericardium and a transmural late gadolinium enhancement involving the mid-segment of the anterior wall and apical segments of both the left and right ventricles suggesting non-viable myocardium (Figure 1*F*–*I*). The right ventricular function was 56% without regional wall motion abnormality and CMR confirmed the severe decrease in LVEF (see Supplementary material  *S2*).

Intramyocardial dissection (ID) is an extremely rare mechanical complication of myocardial infarction,^1^ due to the rupture of intramyocardial vessels causing infiltration of blood into and through the myocardial wall. ID can be contained within the myocardium, known as a contained myocardial rupture or closed ID, or in late stages, and the ID tears into the LV cavity and may cause an open ID, also known as a false pseudoaneurysm.^2^

The patient is currently undergoing medical treatment with functional class III.

This case demonstrates the importance of multimodality cardiac imaging for assessment post-infarction mechanical complications in its different clinical stages.

## Lead author biography


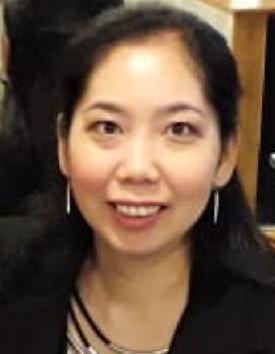


Diana Isabel Katekaru-Tokeshi is a medical doctor graduate from the National Major San Marcos University (Lima, Perú). She got a Cardiology Diploma from the Residency Programme at Hospital Militar Central (Lima, Perú) and fellowship in Cardiac Computed Tomography and Cardiac Magnetic Resonance at “Instituto Nacional de Cardiologia Ignacio Chavez” (Mexico). Now works as attending cardiologist at Hospital Nacional Dos de Mayo (Lima, Perú). Her main fields of interest are congenital heart and ischaemic heart disease.

## Supplementary Material

ytag124_Supplementary_Data

## Data Availability

The data underlying this article will be made available on request to the corresponding author.

## Notes

**Consent:** The authors confirm that written consent for submission and publication of this case report, including images and associated text, has been obtained from the patient in line with COPE guidance.
